# Recent progress of exosomal lncRNA/circRNA–miRNA–mRNA axis in lung cancer: implication for clinical application

**DOI:** 10.3389/fmolb.2024.1417306

**Published:** 2024-07-03

**Authors:** Ren Wang, Yiwei Xu, Liangjing Tong, Xiao Zhang, Sheng Zhang

**Affiliations:** ^1^ Guangzhou Institute of Cancer Research, The Affiliated Cancer Hospital, Guangzhou Medical University, Guangzhou, Guangdong, China; ^2^ Guangzhou Institutes of Biomedicine and Health, Chinese Academy of Sciences, Guangzhou, Guangdong, China; ^3^ GMU-GIBH Joint School of Life Sciences, Guangzhou Medical University, Guangzhou, Guangdong, China

**Keywords:** exosome, non-coding RNAs, lncRNA/circRNA-miRNA-mRNA axis, lung cancer, clinical application

## Abstract

Lung cancer is the leading cause of death among malignant tumors in the world. High lung cancer mortality rate is due to most of patients diagnosed at advanced stage. The Liquid biopsy of lung cancer have received recent interest for early diagnosis. One of the components of liquid biopsy is the exosome. The exosome cargos non-coding-RNAs, especially long non-coding RNAs (lncRNAs), circular RNAs (circRNAs), and microRNAs (miRNAs). The lung cancer derived exosomal non-coding RNAs play the pivotal roles of lung cancer in carcinogenesis, diagnosis, therapy, drug resistance and prognosis of lung cancer. Given ceRNA (competitive endogenous RNA) mechanism, lncRNA or circRNA can act as ceRNA to compete to bind miRNAs and alter the expression of the targeted mRNA, contributing to the development and progression of lung cancer. The current research progress of the roles of the exosomal non-coding-RNAs and the interplay of ceRNAs and miRNAs in mediated lung cancer is illustrated in this article. Hence, we presented an experimentally validated lung cancer derived exosomal non-coding RNAs-regulated target gene axis from already existed evidence in lung cancer. Then LncRNA/circRNA-miRNA-mRNA axis may be a potential target for lung cancer treatment and has great potential in the diagnosis and prognosis of lung cancer.

## 1 Introduction

Lung cancer stands as the most prevalent malignancies globally in 2022, responsible for more than 1.8 million death (Bray et al., 2024). The mortality rate associated with lung cancer is notably high, primarily due to delayed detection and diagnosis, resulting in reduced overall survival rates. Patients with early-stage lung cancer have significantly better prognosis than those with advanced disease (Li et al., 2023). The lung cancer can be classified into two main types: small cell lung cancer (SCLC) and non-small cell lung cancer (NSCLC), representing 15% and 85% of cases respectively (Zhang Y. et al., 2023). The 5-year survival rate for early-stage NSCLC (1A1) can reach 90% (Memon et al., 2024), and even early-stage SCLC patients can achieve a 5-year survival rate of 30% (Liu Q. et al., 2024). However, for patients with advanced-stage lung cancer, the 5-year survival rate dramatically drops to 10%. The absence of clear early symptoms and reliable biomarkers often leads to the late-stage diagnosis of lung cancer, resulting in poorer prognosis. Despite notable advancements in the diagnosis and treatment of lung cancer, the 5-year survival rate remains only 17.9%. This rate further declines to only 4% for patients with distant metastasis (Liao et al., 2023). Therefore, it is crucial to identify new biomarkers to enhance diagnostic accuracy and discover effective therapeutic targets that can facilitate precision treatment.

Tumor biomarkers play a crucial role in the early diagnosis, treatment, therapeutic monitoring and prognosis prediction of lung cancer. Traditional diagnostic methods for early detection of lung cancer include CT and tissue biopsy. However, the CT have limitations such as false positives and radiation exposure, while tissue biopsy are invasive and restricted by tumor heterogeneity (Ettinger et al., 2022). In contrast, liquid biopsy involves monitoring tumors through blood or bronchoalveolar lavage fluid. It offers the advantages of non-invasiveness and bypassing tumor heterogeneity, making it a potentially effective method for early lung cancer diagnosis (Chabon et al., 2020). Currently, the most commonly detected biomarkers in liquid biopsy are circulating tumor cells (CTCs), circulating tumor DNAs (ctDNAs) and exosomes (Casagrande et al., 2023).

Exosomes are small extracellular vesicles with diameters ranging from 30 to 150 nm that are secreted by most cells including the tumor cells through the endoplasmic reticulum pathway (Kalluri and LeBleu, 2020). Exosomes are released through exocytosis once multivesicular bodies fuse with the cell membrane. Exosomes can be isolated from various body fluids, including saliva, blood, pleural effusion, bronchoalveolar lavage fluid, and sputum (Ren et al., 2024). Common methods for exosome isolation comprise ultracentrifugation, ultrafiltration (UF), immune affinity capture and commercial kits like the exoEasy Maxi kit (Qiagen) (Ma et al., 2024a). Exosomes play a role in removing surplus or unnecessary components from cells to maintain cellular homeostasis. Furthermore, they act as vehicles for intercellular substance and information exchange, facilitating communication between tumor cells and nearby or distant cells, as well as stromal cells (Jacobsen Skanderup et al., 2023). Exosomes derived from lung cancer can be obtained from plasma, serum, pleural effusion or bronchoalveolar lavage fluid. Notably, Exosomes secreted by lung cancer cells are more abundant than those secreted by non-tumor cells, making them potential biomarkers for liquid biopsy in lung cancer diagnosis (Choi et al., 2019).

In recent years, an increasing number of studies have indicated that exosome carrying non-coding RNAs (ncRNAs) hold potential as therapeutic targets for lung cancer and serve as new biomarkers for its diagnosis and prognosis (Lai et al., 2023). The dysregulated expression of these ncRNAs within exosome play an important role in driving the development and progression of lung cancer. These vesicles transport essential information, including proteins, lipids and nucleic acids (www.exocarta.org), with a particular focus on ncRNAs (miRNAs, lncRNAs and circRNAs), which elevates their significance (Clancy and D'Souz-Schorey, 2023). Exosome not only serves vital roles in intercellular material and information transfer but also exhibit close associations with tumor development and progression, making them promising early diagnostic markers for various cancers. Sequencing of exosomal RNA enables rapid and efficient acquisition of comprehensive information, rendering it an ideal approach for disease diagnosis and prognosis (Gupta, 2022). Current research on ncRNAs in exosome predominantly centers on the significant regulatory roles of miRNA, lncRNA and circRNA in transcription, post-transcription and translation, positioning them as potential biological targets for the prevention, diagnosis and treatment of lung cancer (Zhao et al., 2023).

**FIGURE 1:**
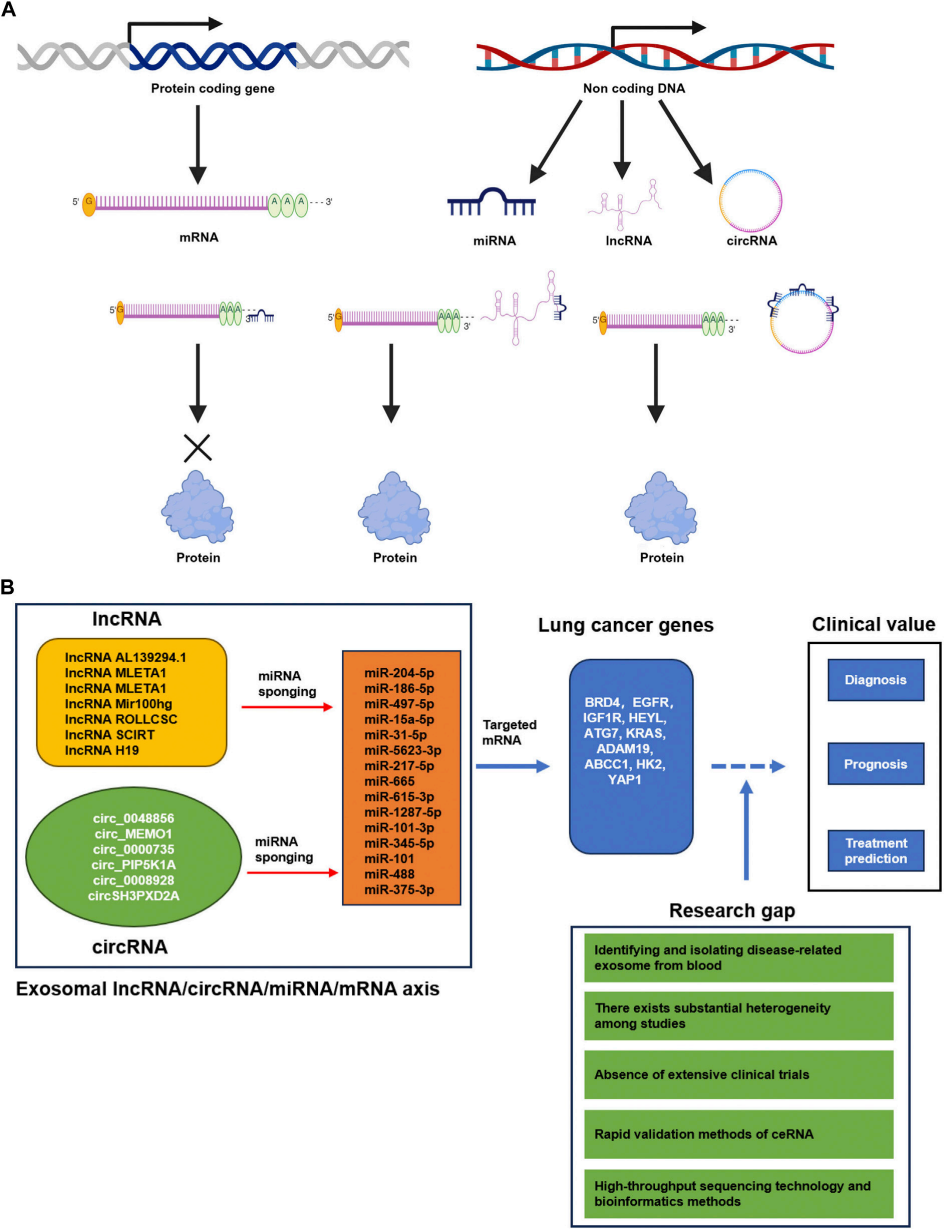
**(A)** The CeRNA mechanism (Created with BioRender.com). Protein-coding genes undergo transcription, resulting in the production of mRNA that is translated to proteins. Non-coding DNA is transcribed into various types of ncRNAs, including miRNA, lncRNA, and circRNA. These ncRNAs play an important role in regulating mRNA translation. Specifically, miRNAs bind to partially complementary sequences located in either the 3′UTR or ORF regions of target mRNAs, thereby impeding their translation. These partially complementary sequences are denoted as microRNA response elements (MREs). Likewise, lncRNAs and circRNAs also harbor numerous MREs, and they competitively bind to miRNAs, acting as sponges, ultimately enhancing mRNA translation into proteins. These ncRNAs that engage in competitive interactions are referred to as competing endogenous RNAs (ceRNAs). **(B)** Summary of the current potential lncRNA/circRNA-miRNA-mRNA axis derived from lung cancer exosomes in diagnosis, prognosis and treatment prediction of lung cancer. The GAP of basic research and clinical application in lung cancer.

The miRNA refers to endogenous ncRNA molecules approximately 18–22 nucleotides in size, capable of binding to complementary sequences in the 3′untranslated region (UTR) of target gene’s mRNA through miRNA response elements (MRE). This binding inhibits the expression of protein-coding genes and leads to the renewal, conversion or degradation of mRNA transcripts (Kilikevicius et al., 2022). In 2011, Salmena et al. proposed the hypothesis of a competitive endogenous RNA (ceRNA) interaction mechanism between non-coding RNA and mRNA (Salmena et al., 2011). According to this hypothesis, the lncRNA and circRNA containing MREs can alleviate the inhibitory effect of miRNA on target genes by binding to miRNA (Figure 1A). Consequently, the ceRNA can regulate the translation of protein encoded by specific mRNA by modify their binding with miRNAs through the sponge effect. In recent years, numerous studies have focused on the investigation of ncRNAs in relation to lung cancer. Specifically, the intricate crosstalk between various CeRNAs and miRNA has emerged as a promising avenue for both therapeutic targeting and biomarker development in lung cancer (Wu X. et al., 2020). This review aims to provide an overview and discussion of the potential roles of three types of exosomal ncRNAs in liquid biopsy, with specific emphasis on their applications in the diagnosis, therapy and prognosis of lung cancer. Moreover, highlight the hope of the lncRNA/circRNA-miRNA-mRNA axis for reliable identification, prediction and therapy of lung cancer.

## 2 Exosomal miRNAs as potential biomarkers for the treatment of lung cancer

Exosomes, along with the enclosed miRNA, are taken up by neighboring or distant cells, regulating the translation of target mRNA and thereby modulating processes related to tumor immunity and the tumor microenvironment. This modulation has the potential to facilitate tumor growth, invasion, metastasis, angiogenesis and drug resistance. Consequently, the role of exosomal miRNA in the regulation of cancer progression is substantial, making them promising candidates as diagnostic and therapeutic biomarkers (Sun et al., 2018). Additionally, besides their involvement in tumors, evidence indicates that exosome miRNA is implicated in corneal diseases (Verma et al., 2023; Arora and Verma, 2024), complications of diabetes (Xu et al., 2022), and autoimmune skin diseases (Zhang R. et al., 2023). Hence, they possess the potential to serve as biomarkers and therapeutic targets in these conditions. Recent studies have shown that the exosomal secretion of miRNA has potential as an ideal diagnostic biomarker for the clinical application of lung cancer. Certain exosomal miRNAs found in plasma or serum exhibit high expression levels in NSCLC, thus serving as specific biomarkers for its diagnosis and prognosis (Table 1). Notably, the exosomal miR-146a-5p/miR-486-5p (Wu Q. et al., 2020), miR-619-5p/miR-4454 (Feng et al., 2023), miR-21/miR-25/miR-155/miR-210/miR-486 (Liu et al., 2020) and EV-miR-486-5p/miR-21-5p (Tong et al., 2024) were upregulated in NSCLC patients than in healthy people. The high expression of these three panels EV-miRNAs is positive correlation with NSCLC diagnosis. Cazzoli et al. observed higher expression levels of four miRNAs (miR-378A/miR-379/miR-139-5P/miR−200B-5P) in exosomes extracted from the blood of patients with lung adenocarcinoma compared to healthy smokers in 2013 (Cazzoli et al., 2013). Additionally, mi1290/miR-29C-3p (Zhang Q. et al., 2023) and miR-7977 (Chen et al., 2020) showed elevated expression levels in exosomes and served as effective diagnostic biomarkers for lung adenocarcinoma. These miRNAs present promising candidates for the development of highly sensitive and non-invasive early diagnostic biomarkers for NSCLC.

**TABLE 1:** Exosomal ncRNAs as potential diagnostic and prognostic biomarkers in lung cancer.

Exosomal ncRNAs		Biomarker potential	Origin of exosome	Status	Clinical evidence	Cition
Exosomal miRNAs as potential diagnostic and prognostic biomarkers in lung cancer						
miR-146a-5p/miR-486-5p		diagnostic	NSCLC Serum	Upregulated	YES	Wu Q. et al. (2020)
miR-21, miR-25, miR-155, miR-210/miR-486		diagnostic	NSCLC Serum	Upregulated	YES	Liu et al. (2020)
miR-486-5p/miR-21-5p		diagnostic	NSCLC Serum	Upregulated	YES	Tong et al. (2024)
miR-378A, miR-379, miR-139-5P/miR-200B-5P		diagnostic	Adenocarcinoma Plasma	upregulated	YES	Cazzoli et al. (2013)
miR-7977		diagnostic	Adenocarcinoma Serum	Upregulated	NO	Chen et al. (2020)
mi1290/miR-29C-3p		diagnostic	SCC Plasma	Upregulated	YES	Zhang Q. et al. (2023)
miR-619-5p/miR-4454		diagnostic	Adenocarcinoma Plasma	Upregulated	NO	Feng et al. (2023)
miR-1290		prognostic	Adenocarcinoma Plasma	Upregulated DFS	YES	Wu Y. et al. (2020)
miR4497		prognostic	NSCLC Plasma	Upregulated DFS	YES	Zheng et al. (2023)
mir-451 a		prognostic	NSCLC Plasma	Upregulated	YES	Kanaoka et al. (2018)
Exosomal lncRNAs as potential diagnostic and prognostic marker in lung cancer						
TBILA and AGAP2-AS1	Diagnostic	NSCLC Serum		Upregulated	YES	Tao et al. (2020)
DLX6-AS1		Diagnostic	NSCLC Serum	Upregulated	NO	Zhang et al. (2019)
SLC9A3-AS1 and PCAT6		Diagnostic	lung cancer	Upregulated	NO	Bai et al. (2019)
GAS5	diagnostic	early NSCLC. serum		Downregulated	YES	Li et al. (2019)
Lnc-RNA-RP11-510M2	Diagnostic	lung cancer serum		Downregulated	NO	Mohamed Gamal El-Din et al. (2022)
LncCRLA	diagnostic	Adenocarcinoma plasma		Upregulated	YES	Fan et al. (2020)
SOX2-OT	diagnostic	SCC plasma		Upregulated	YES	Teng et al. (2019)
RP5-977B1	diagnostic and prognostic	NSCLC Serum		Upregulated	YES	Min et al. (2022)
HAGLR	prognostic	NSCLC Serum		Upregulated	NO	Le et al. (2019)
linc01125	Diagnostic, prognostic	NSCLC Serum		Upregulated	NO	Xian et al. (2021)
LINC00917	Diagnostic, prognostic	NSCLC Serum		Upregulated	NO	Xiong et al. (2021)
Exosomal circRNAs as potential diagnostic and prognostic marker in lung cancer						
circ_0047921, circ_0056285, circ_0007761		Diagnostic	NSCLC BLOOD	Upregulated	YES	Xian et al. (2020)
circ_0001439, circ_0001492, circ_0000896		Diagnostic	Adenocarcinoma Serum	Upregulated	YES	Kang et al. (2022)

Exosomal miRNAs not only served as diagnostic markers but also function as valuable prognostic indicators for lung cancer patients. The level of miR-4497 (Zheng et al., 2023), miR-1290 (Wu Y. et al., 2020), and miR-133a-3p (Yang et al., 2023) are all significantly and inversely associated with patients’ progression-free survival (PFS). Moreover, miR-451a (Kanaoka et al., 2018) also reliably predicts OS in NSCLC. These exosomal miRNAs play a crucial role in lung cancer prognosis.

## 3 Exosomal LncRNAs as potential biomarkers for the treatment of lung cancer

LncRNAs are typically characterized as non-coding transcripts exceeding 200 nucleotides in length, which regulate protein expression at various levels (Herman et al., 2022). Increasing evidences confirms the distinct expression patterns of lncRNAs in tumor tissues and tumor-derived exosomes. Significantly, exosomal lncRNAs can serve as potential biomarkers for identification the lung cancer (Table 1). Notably, exosomal lncRNAs TBILA/AGAP2-AS1 (Tao et al., 2020) and DLX6-AS1 (Zhang et al., 2019) were found to be upregulated in NSCLC. They exhibited significant positive correlations with the tumor size, lymph node metastasis and TNM stage of the NSCLC, thus serving as diagnostic markers for NSCLC. Furthermore, exosomal lncRNA SLC9A3-AS1/PCAT6 showed elevated expression in lung cancer patients and can potentially be employed for identifying lung cancer (Bai et al., 2019). Additionally, the lncRNA GAS5 (Li et al., 2019) and Lnc-RNA-RP11-510M2 (Mohamed Gamal El-Din et al., 2022) were observed to be expressed at lower levels in NSCLC and lung cancer compared to healthy controls respectively, also making them potential biomarkers for distinguishing lung cancer. Furthermore, lncCRLA exhibited specific expression in adenocarcinoma cells, mouse adenocarcinoma tissues and patient plasma exosomes, rendering it valuable for predicting the occurrence of lung adenocarcinoma (Fan et al., 2020). Notably, in lung SCC, the exosomal lncRNA SOX2-OT displayed significant upregulation when compared to exosomes from healthy individuals. The findings demonstrated a positive correlation between exosomal SOX2-OT levels and tumor size, TNM staging and lymph node metastasis, thereby enabling the detection of SCC (Teng et al., 2019). Additionally, exosomal lncRNA RP5-977B1 exhibited higher expression levels in NSCLC compared to the healthy control group. This aberrant expression was associated with the diagnosis of NSCLC and a shorter OS (Min et al., 2022). Subsequent research revealed that lnc00917 (Xiong et al., 2021), lnc01125 (Xian et al., 2021) and HAGLR (Le et al., 2019) were all highly expressed in NSCLC patients and demonstrated positive correlations with tumor stage and lymph node metastasis. Consequently, they have emerged as potential candidate biomarkers for diagnosing and predicting the prognosis of NSCLC.

## 4 Exosomal circRNAs as potential biomarkers for the treatment of lung cancer

CircRNAs are a rapidly growing subgroup of non-coding RNA molecules, which have captured considerable attention because of their implicated roles in oncogenesis. The growing researches have suggested that exosomal circRNAs can serve as vital diagnostic and prognostic biomarkers for different types of lung cancer (Table 1) (Zhang F. et al., 2023). Notably the synergistic utilization of circ_0047921/circ_0056285/circ_0007761 has demonstrated a substantial improvement in the diagnostic accuracy for NSCLC (Xian et al., 2020). Current investigations have also shown that the levels of serum exosomal circ_0001492/circ_0001439/circ_0000896 are significantly elevated in individuals with lung adenocarcinoma compared to healthy subjects, and their expression levels is experienced a pronounced decrease after surgical intervention. Moreover, the combination of these three specific serum exosomal circRNAs has exhibited superior efficacy, heightened sensitivity and specificity in the detection of lung adenocarcinoma (Kang et al., 2022). Based on these evidences, exosomal circRNAs are emerging as exceptionally promising and reliable biomarkers for the detection and prognosis of lung cancer.

## 5 Lung cancer exosomal lncRNA/circRNA–miRNA–mRNA as potential biomarkers

These exosomal ncRNAs (miRNAs, lncRNA and circRNA) play an important role in regulating mRNA translation. Specifically, miRNAs bind to partially complementary sequences located in either the 3′UTR or ORF regions of target mRNAs, thereby impeding their translation. These partially complementary sequences are denoted as microRNA response elements (MREs). Likewise, lncRNAs and circRNAs also harbor numerous MREs, and they competitively bind to miRNAs, acting as sponges, ultimately enhancing mRNA translation into proteins. These ncRNAs that engage in competitive interactions are referred to as competing endogenous RNAs (ceRNAs). Exosomes secreted by lung cancer cells can transport the cancer cell secreted lncRNAs or ciricRNAs to nearby or distant cells, as well as stromal cells. And then the lncRNAs or ciricRNAs can upregulated the targeted gene expression by the ceRNA mechnisms in these cells. Finaly, the ceRNA can influence tumor progression and tumor microenvironment. Exosomal lncRNA/circRNA-miRNA-mRNA regulatory networks have the ability to upregulate target gene expression and effectively regulate the progression of lung cancer (Salmena et al., 2011). Thus, the exosomal lncRNA/circRNA–miRNA–mRNA axis holds significant potential as a diagnostic and identification tool for lung cancer. For instance, recent studies have demonstrated that lncRNA AL139294.1 was transported to recipient cells through exosome. LncRNA AL139294.1 promotes lung cancer metastasis *in vitro* and *in vivo* by competitively binding with miR-204-5p to regulate BRD4 and activate the Wnt and NF-κB2 pathways. Moreover, the lncRNA AL139294.1 is upregulated in the exosomes of NSCLC patients’ serum, while miR-204-5p is downregulated. The increased level of lncRNA AL139294.1 and decreased level of miR-204-5p are positively correlated with advanced pathological stage, lymph node metastasis and distant metastasis in NSCLC patients (Ma et al., 2024b). Thus, the AL139294.1-miR-204-5p-BRD4 axis has the potential to serve as a diagnostic biomarker for non-small cell lung cancer. Similarly, exosomal lncRNA MLETA1miR-186-5p-EGFR and MLETA1-miR-497-5p-IGF1R axes have been found to promote lung cancer metastasis. And then their expression within exosomes negatively correlates with the non-metastatic survival of patients, making them as prognostic biomarkers and potential therapeutic targets for lung cancer (Hsu et al., 2023). Shi Q. et al. (2023) demonstrated that the exosomal transport of lncRNA Mir100 hg enhances glycolysis and promotes lung adenocarcinoma metastasis through interaction with miRNA-15a-5p/31-5p (Shi L. et al., 2023). Zhang et al. (2024) found that exosomal lncRNA ROLLCSC stimulates lipid metabolism and promotes lung cancer metastasis through its ceRNA function with miR-5623-3p and miR-217-5p (Zhang et al., 2024). Additionally, these exosomal lncRNA ceRNA regulatory networks may serve as biomarkers for DFS. Furthermore, the transfer of ncRNA H19 through exosomes has been found to promote resistance to Erlotinib in non-small cell lung cancer via the miR-615-3p-ATG7 axis, and it can potentially predict the efficacy of Erlotinib treatment (Pan and Zhou, 2020).

Exosomal circRNAs have been implicated in ceRNA regulatory networks associated with the progression of lung cancer. Specifically, Circ-MEMO1 (Ding et al., 2020), circ_0048856 (He et al., 2022), and circ_0000735 (Liu S. et al., 2024) derived from exosomes exhibit increased levels in the serum and cell lines of NSCLC. Exosomal circ_0048856-miR-1287-5p, circ-MEMO1-miR-101-3p-KRAS axis, and circ_0000735by-miR-345-5p-ADAM19 pathway promotes NSCLC development. These regulatory networks encompassing circ_0048856-miR-1287-5p, circ-MEMO1-miR-101-3p-KRAS, and circ_00007355-miR-345-5p-ADAM19 play a role of detection of non-small cell lung cancer. Additionally, silencing exosomal circ_PIP5K1A suppresses NSCLC progression and enhances cisplatin sensitivity through modulation of the miR-101-ABCC1 axis (Shao et al., 2021), whereas knockdown of exosomal circ_0008928 inhibits NSCLC progression and augments cisplatin sensitivity via regulation of the miR-488-HK2 axis (Shi Q. et al., 2023). Moreover, exosomal circSH3PXD2A mitigates DDP chemotherapy resistance in SCLC by modulating the miR-375-3p-YAP1 axis (Chao et al., 2023). These discoveries introduce novel potential biomarkers for predicting the efficacy of drug resistance treatment in both non-small cell lung cancer and SCLC.

## 6 Conclusion

Studying non-invasive and effective methods for the detection of lung cancer has significant importance in reducing the risk of death in patients with this disease. The research on exosome ncRNA has made continuous progress, and its functional role as stable and non-invasive biomarkers for the diagnosis, prognosis, and treatment response of various cancers, especially lung cancer, has garnered much attention. In this review, we primarily summarize the clinical value of exosome ncRNA, including miRNA, lncRNA, circRNA and the complex crosslink of ceRNA, in the diagnosis, prognosis, and treatment prediction of lung cancers (Figure 1B). The study of exosome ceRNA complex crosslink in cancer research is an emerging field, and advancements in understanding the role of exosomal lncRNA/circRNA-miRNA-mRNA axis in lung cancer have been made through basic research. There is mounting evidence to suggests that exosomal ncRNA biomarkers have prospective applications in the diagnosis and prognosis of lung cancer. However, the clinical use of exosomal noncoding RNA biomarkers in lung cancer patients is still in the preclinical stage, and there are certain obstacles in clinical translation. The present challenge in the field revolves around the efficient isolation and identification of disease-related exosomes from blood. Additionally, there is a need to identify specific ncRNAs, such as miRNA, lncRNA, circRNA and ceRNA, as potential early diagnostic markers derived from disease-related exosomes. Furthermore, precise cut-off values are required to identify specific ncRNAs, including miRNA, lncRNA, circRNA and ceRNA, as potential early diagnostic markers derived from disease-related Exosomes. Moreover, there exists substantial heterogeneity among studies. Additionally, the absence of extensive clinical trials hinders the confirmation of their potential value (Li et al., 2021). The development of high-throughput sequencing technology and bioinformatics methods such as whole exosome sequencing (WES) will enable future studies to compare and analyze the exosomal ncRNA and lncRNA/circRNA-miRNA-mRNA of lung cancer tissues under various physiological and pathological conditions, providing a global understanding of exosomal lncRNA/circRNA-miRNA-mRNA axis (George et al., 2024). This comprehensive understanding will contribute to the discovery of novel exosomal lncRNA/circRNA-miRNA-mRNA axis. Furthermore, it is crucial to explore rapid validation methods and decipher the mechanisms of lncRNA/circRNA-miRNA-mRNA axis in lung cancer development. Currently, there is limited research on the clinical application of lncRNA/circRNA-miRNA-mRNA, and further studies are needed to demonstrate their clinical value (Figure 1B). Hopefully, in the near future, non-invasive detection of exosomal ncRNA can be implemented for the screening of lung cancer patients.
